# Enhancing soil organic carbon, particulate organic carbon and microbial biomass in semi-arid rangeland using pasture enclosures

**DOI:** 10.1186/s12898-018-0202-z

**Published:** 2018-11-06

**Authors:** C. O. Oduor, N. K. Karanja, R. N. Onwonga, S. M. Mureithi, D. Pelster, G. Nyberg

**Affiliations:** 1 Department of Land Resource Management and Agricultural Technology (LARMAT), University of Nairobi, P. O. Box 29053-00625, Nairobi, Kenya; 2 Mazingira Centre, International Livestock Research Institute, P. O. Box 30709–00100, Nairobi, Kenya; 3 Agriculture and Agri-Food Canada, Science and Technology Branch, Quebec City, Canada; 4 Department of Forest Ecology and Management, Swedish University of Agricultural Sciences (SLU), 90183 Umeå, Sweden

**Keywords:** Enclosure, Environmental microbiology, Land degradation, Microbial biomass, Organic carbon, Particulate carbon, Rehabilitation, Soil quality

## Abstract

**Background:**

Rehabilitation of degraded rangelands through the establishment of enclosures (fencing grazing lands) is believed to improve soil quality and livelihoods, and enhance the sustainability of rangelands. Grazing dominated enclosure (GDE) and contractual grazing enclosure (CGE) are the common enclosure management systems in West Pokot County, Kenya. Under CGE, a farmer owning few animals leases the enclosure to households with relatively more livestock, while GDE is where the livestock utilizing the enclosure are purely owned by the farmer. Livestock management in both systems is via the free-range system. This study evaluated the effect of enclosure management on total soil organic carbon (SOC), particulate organic carbon (POC) and microbial biomass carbon (MBC) and nitrogen (MBN) as key indicators of soil degradation at 0–40 cm depth. The two enclosure systems were selected based on three age classes (3–10, 11–20 and > 20 years since establishment) (n = 3). The adjacent open grazing area (OGR) was used as a reference (n = 9).

**Results:**

Relative to OGR, the pasture enclosures significantly decreased soil bulk density and increased the concentrations of total organic C, POC, MBC and MBN compared to the degraded OGR (*P* < 0.001). Significantly higher concentrations of POC and MBC was recorded in GDE than CGE (*P *= 0.01). The POC accounted for 24.5–29.5% of the total SOC. MBC concentrations ranged from 32.05 ± 7.25 to 96.63 ± 5.31 µg C g^−1^ of soil in all grazing systems, and was positively correlated with total SOC and POC (*P* < 0.001). The proportional increase in POC and MBC was higher in GDE (56.6 and 30.5% respectively) compared to CGE (39.2 and 13.9% for POC and MBC respectively).

**Conclusions:**

This study demonstrated that controlling livestock grazing through the establishment of pasture enclosures is the key strategy to enhance total SOC, POC, MBC, and MBN in degraded rangelands; a precondition for improving soil quality. Therefore, the establishment of enclosures is an effective restoration approach to restore degraded soils in semi-arid rangelands.

## Background

Human-induced soil degradation is a major concern globally [1, 2], and has contributed to the decline in net primary productivity in arid and semi-arid lands [3]. Overgrazing in rangelands has altered the natural ecosystem, causing disturbances in biotic and abiotic components and livelihood of the community. Among the negative impacts of overgrazing is the loss of soil organic carbon (SOC), a scenario that occurs both in the temperate [4–6] and tropical rangelands [7, 8]. Soil organic carbon is the basis for soil fertility, the source of energy for soil microorganisms and regulates climate and biodiversity [9–12]. Restoration degraded rangelands have therefore attracted considerable attention in the recent past. The restoration of degraded grazing land may be important to improve the accumulation of SOC soil. The establishment of pasture enclosures by fencing degraded communal grazing areas has been reported to reduce the negative impacts of continuous grazing by preserving soil resources, leading to accumulation of SOC that was previously lost [7, 12, 13]. Understanding the dynamics and potential of soil to store organic carbon is not only essential for improving soil quality and enhancing the sustainability of rangelands in Sub-Saharan Africa, but also mitigate climate change by offsetting CO_2_ emissions [4, 14].

Soil organic carbon is regarded as an indicator of soil quality and by extension, the state of soil degradation as it determines soil structure, nutrient retention and supports biological diversity [15–17]. The reduction or loss of SOC could, therefore, lower soil fertility and consequently, lead to land degradation [18]. According to [13], the establishment of enclosure in a degraded rangeland resulted to a 34% increase in total SOC content in the upper 40 cm layer of soil. Besides, [19] recounted that degraded soils in semi-arid rangeland with low levels of organic carbon may be functionally improved by establishing pasture enclosures. However, [14] and [15] acknowledged that changes in total SOC require several years to detect. The labile fractions of total SOC include particulate organic carbon (POC) and microbial biomass carbon (MBC) [20]. These fractions may be more sensitive to land management than the total SOC. The POC acts as a substrate for soil microorganisms and influences soil nutrient cycles and biological properties of soil [21].

Livestock enclosures have gained cognizance as a successful tool for controlling heavy grazing and land degradation in Eastern Africa [19, 22–24]. In the arid and semi-arid rangelands of Western Kenya, efforts to restore degraded grazing lands through the establishment of pasture enclosures started in the mid-1980s [25]. As indicated by [26], grazing dominated enclosure (GDE) and contractual grazing enclosure (CGE) are the common types of enclosure management systems in West Pokot County, Kenya. The enclosures are privately owned and utilized, with an average size of 5 ha [26]. Contractual grazing represents a grazing arrangement where a farmer owning few animals leases the enclosure to households with relatively more livestock. On the other hand, GDE is where the livestock utilizing the enclosure is purely owned by the farmer. The stocking rate of the enclosures in the area ranges between 1 and 42 animals with a mean of 7 animals [26]. Livestock management in both CGE and GDE systems is via the free-range system. Previous studies in semi-arid rangelands show that POC and MBC concentrations increase after enclosing degraded grazing lands [6, 19, 27], while others reported that grazing management has no significant impact on the dynamics of labile fractions of carbon [28, 29]. These variations were attributed to differences in soils [30] and vegetation characteristics such as litter quantity and quality [31–33]. However, pasture management in the former studies was via cut-and-curry where livestock was not allowed to graze (excluded) in the enclosures.

Despite the fact that the practice of enclosures has existed in West Pokot County for over three decades, data on the effectiveness of these enclosures to restore degraded soils in terms of organic C in the area is lacking. Understanding the effect of enclosure management system and their age on SOC is crucial to offer the most effective carbon management options in rangelands. Based on the hypothesis that GDE enclosures are more effective to restore degraded soils than CGE enclosures by improving the content of soil organic carbon and microbial biomass, this study was carried out to determine the concentrations of total SOC, POC and MBC in CGE and GDE under three age-classes (3–10, 10–20, and > 20 years since effective protection) with the similar quantifications in the adjacent open grazing areas as the baseline.

## Methods

### Study site

The study was conducted in Chepareria Ward (01°18′17″–01°19′41″N and 035°14′16″–035°15′49″E, 1680 m. a. s. l) in West Pokot County, Northwestern Kenya. The area is classified as semi-arid; receiving an average rainfall of 280 mm of rainfall for the short rains which occur between mid-October and January and 570 mm for the long rains which occur from mid-March to July [34]. The annual average daily air temperature ranges between 16 and 30 °C [34]. The soils are predominantly sandy clay to loamy sand and are classified as Haplic Lixisols [35]. Vegetation is predominantly grassland (*Themeda triandra*, *Eragrostis superba*, *Cymbopogon validus*, *Cenchrus ciliaris* and *Cynodon dactylon*) [36], with scattered native *(Acacia* spp., *Balanites aegyptiaca*, and *Kigelia africana*) and exotic (*Grevillea robusta*) tree species [22]. The average herbaceous vegetation cover range between 20.7% in open grazing rangeland and 40.2% in enclosure systems, with 72.0 kg dry matter (DM) ha^−1^ and 521.8 kg DM ha^−1^ of herbaceous above-ground biomass in open grazing rangeland and enclosure respectively [36]. The traditional open grazing areas are characterized by free-range grazing of livestock with a stocking rate that exceeds the upper limit of the enclosure systems. The open grazing areas had a history of severe land degradation prior to the establishment of enclosures in mid-1980s by Vi-Agroforestry [25].

### Soil sampling

Soil sampling was carried out during the short rain season, November 2016. In consultation with the local leaders and Vi-Agroforestry officials, the CGE and GDE enclosures were grouped into three age classes: 3–10 years, 11–20 years, and > 20 years, and three enclosures were randomly selected from each enclosure type/age class combination. A total of 18 enclosures were selected for sampling. Nine open grazing sites (OGR) were selected as controls (n = 9). This gave a total of 27 sampling sites. Within each enclosure/age class and in the adjacent open grazing areas, three 50-m transects were laid out in a Z-shaped orientation, at least 10 m from the edge to avoid edge effects. Along each transect, five sampling points were laid at 10 m apart and soil samples collected using a soil auger at 0–10, 10–20, and 20–40 cm depths. The five soil samples at each depth and within each transect were mixed to form a composite sample, producing three composite samples (one for each depth) for each transect and a total of nine composite samples (3 depths × 3 transects) from each enclosure and open grazing site. A total of 243 soil samples were obtained (27 sampling sites × 9 composite samples). About 0.5-kg sub-sample was placed in air tight plastic bags for soil moisture determination, extraction of microbial biomass carbon (MBC) and microbial biomass nitrogen (MBN). The remainder of the soil was air-dried, sieved through a 2-mm mesh and stored at 4 °C in a refrigerator for physical and chemical analyses. Steel cylinders of 98.2 cm^−3^ were used to obtain undisturbed soil samples for soil bulk density determinations, using the same sampling design. Within each transect, a 40 cm profile pit was dug in and one core sample taken in each depth, making a total of three core samples per transect.

### Soil analysis in the laboratory

Soil water content was determined gravimetrically by oven-drying 100 g soil sub-sample at 105 °C to constant weight for 48 h [37]. Soil pH and electrical conductivity (EC) were determined in soil–water suspension in the ratio 1:2.5 (weight/volume). Soil pH was measured using a glass electrode pH meter (model: HI 2211, Hanna instruments), while EC was measured using a conductivity meter (model: HI 9812, Hanna Instruments). Soil bulk density (BD) was determined using core ring method by oven-drying core samples at 105 °C for 48 h [38], and particle size distribution using the hydrometer method [39]. Total soil organic carbon (SOC) was determined using the wet oxidation method [40], total nitrogen (TN) was determined using the Kjeldahl method [41] and cation exchange capacity (CEC) was determined by the ammonium acetate (NH_4_OAc) method as described by [42].

Physical fractionation was used to determine particulate organic carbon content, associated with the sand fraction (2000–53 μm), following procedures by Cambardella and Elliott [43]. Approximately 20-g of sieved (< 2.0 mm) air-dried soil sub-sample was dispersed with 70 ml of 5-g l^−1^ sodium hexametaphosphate solution and the suspension was passed through a 53 μm sieve using a jet distilled water. The material retained in the sieve was dried at 45 °C for 48 h in a forced air oven. The oven-dried material was ground and analyzed for organic carbon by the wet oxidation method [40] and TN using the Kjeldahl method [41].

Chloroform fumigation-extraction method was used to determine MBC and MBN contents in soil [44]. Ethanol-free chloroform was used to fumigate 10 g of field-moist soil samples for 24 h in a vacuum desiccator at room temperature. Another set of the same soil samples were not fumigated. The soluble C from the fumigated and non-fumigated samples was extracted with 50 ml of 0.5-M K_2_SO_4_ solution. The extracted soil MBC was measured spectrophotometrically at 600 nm. The difference between the extracted C in the fumigated and non-fumigated soils represented the microbial biomass C [45]. MBN was determined by digesting 20 ml of the soil extract using Kjeldahl digestion and the digest analyzed for total N. Correction factors (kc) of 0.45 and 0.54 were used for MBC and MBN respectively [46, 47].

### Statistical analysis

Effects of grazing systems and soil depths, and enclosure type and age on total SOC, SOC fractions, microbial biomass, and the interactions were analyzed by two-way analysis of variance (ANOVA) using Genstat 15th edition [48]. Means were separated using Fischer’s protected least significant difference (LSD) test at *P* ≤ 0.05. Pearson correlation analyses were conducted to establish the relationship between soil organic carbon fractions and soil texture and microbial biomass carbon using SPSS 20th version [49].

## Results

### Soil physical and chemical characteristics

The sand, silt and clay contents were similar for all the grazing systems (Table 1). Soil bulk density in the 0–10 cm was lowered significantly from 1.49 g cm^−3^ in the OGR to 1.42 and 1.39 g cm^−3^ in CGE and GDE enclosures respectively (*P *< 0.001, Table 1). Soil moisture content was generally higher in the enclosures relative to the OGR and increased with depth. The enclosure system did not significantly alter soil pH and CEC (Table 1).

**Table 1:** Soil physical and chemical properties under different grazing management systems in Chepareria, Kenya

Grazing system	Soil depth (cm)	pH	CEC	Sand	Silt	Clay	Moisture content	BD
			Cmol kg^−1^	%				g cm^−3^
GDE	0–10	6.1 ± 0.55	8.0 ± 1.03	78.7 ± 2.61	5.4 ± 1.62	13.6 ± 1.17	6.79 ± 2.27bc	1.39 ± 0.10bc
	10–20	6.1 ± 0.30	8.3 ± 0.93	77.8 ± 2.52	5.7 ± 2.37	14.2 ± 1.09	7.28 ± 2.29abc	1.37 ± 0.05c
	20–40	6.0 ± 0.34	9.1 ± 0.78	78.2 ± 2.52	6.0 ± 2.00	14.0 ± 1.15	8.16 ± 2.23ab	1.36 ± 0.06c
CGE	0–10	6.2 ± 0.22	8.2 ± 0.75	81.3 ± 1.29	7.8 ± 1.60	13.4 ± 1.21	6.32 ± 2.76c	1.42 ± 0.10abc
	10–20	6.0 ± 0.61	8.7 ± 0.95	80.6 ± 1.57	8.0 ± 2.23	13.7 ± 1.16	6.83 ± 2.68bc	1.46 ± 0.10ab
	20–40	6.2 ± 0.24	8.6 ± 1.16	80.6 ± 1.60	7.7 ± 2.88	13.4 ± 1.18	8.51 ± 2.44a	1.45 ± 0.05ab
OGR	0–10	6.3 ± 0.27	9.0 ± 0.92	79.5 ± 1.61	6.8 ± 1.88	13.8 ± 1.29	5.85 ± 2.51c	1.49 ± 0.05a
	10–20	5.2 ± 0.56	8.6 ± 0.95	78. 9 ± 1.57	7.3 ± 2.09	13.7 ± 1.30	6.38 ± 2.55c	1.47 ± 0.06a
	20–40	5.0 ± 0.24	8.7 ± 0.90	78.7 ± 1.48	7.2 ± 1.75	13.9 ± 1.17	6.78 ± 2.22bc	1.47 ± 0.06a
LSD_0.05_		NS	NS	NS	NS	NS	1.13	0.07
cv%		6.6	10.7	2.4	31.8	8.7	15	5.2
P-value		0.13	0.56	0.16	0.08	0.168	0.01	< 0.001

Values are mean ± SD (n = 9). Different lowercase letters within the same column indicate significant differences between means at *P *≤ 0.05

*NS* not significant, *GDE* grazing dominated enclosure, *CGE* contractual grazing enclosure, *OGR* open grazing rangeland, *CV%* coefficient of variation

### Total soil organic carbon and nitrogen

Grazing system and soil depth had significant (*P *< 0.001) effect on total SOC concentration. The proportion total SOC in the enclosures was 27.1% higher compared to OGR and the concentration decreased with depth (Table 2). However, the difference in SOC content between CGE and GDE was not significant. On the other hand, the values of total N content in CGE and GDE were similar but highly significant (*P *< 0.001) compared to total N content in OGR (Table 2). Within the enclosure systems, the age of enclosure had no effect on total SOC and TN concentrations (*P *= 0.52).

**Table 2:** Soil organic carbon and total nitrogen concentrations at three depths under different grazing management systems

Depth (cm)	Total soil organic carbon (g kg^−1^)			Total nitrogen (g kg^−1^)		
	OGR	CGE	GDE	OGR	CGE	GDE
0–10	4.93 ± 0.69Ba	6.22 ± 0.78Aa	6.61 ± 0.89Aa	0.53 ± 0.07Ba	0.63 ± 0.08Aa	0.65 ± 0.08Aa
10–20	4.88 ± 0.65Ba	5.86 ± 0.67Aa	6.28 ± 0.99Aa	0.58 ± 0.11Ba	0.63 ± 0.08Aa	0.61 ± 0.07ABa
20–40	4.36 ± 0.74Bb	5.57 ± 0.57Ab	5.47 ± 0.77Ab	0.52 ± 0.10Bb	0.61 ± 0.08Aa	0.59 ± 0.07Ab
Pooled mean	4.72 ± 0.73B	5.88 ± 0.72A	6.12 ± 1.00A	0.54 ± 0.09B	0.62 ± 0.08A	0.62 ± 0.08A

Values are mean ± SD (n = 9). Values with different uppercase letters across the rows (grazing systems) and the lowercase letters within columns (soil depths) are significantly different at *P* < 0.05

*OGR* Open grazing rangeland, *CGE* contractual grazing enclosure, *GDE* grazing dominated enclosure

### Particulate organic carbon

Grazing management significantly affected the concentration of POC (Table 3). The concentration of POC in the 0–10 cm increased significantly (*P* < 0.001) from 1.40 ± 0.21 in OGR to 2.01 ± 0.26 in CGE and 2.28 ± 0.34 g kg^−1^ in GDE (Table 3). Unlike total SOC, the difference in POC content between CGE and GDE was significant (*P *= 0.01), but exhibited no significant variations among the age classes (*P *= 0.71). Relative to OGR, the proportion of POC in CGE and GDE was high by 38.8 and 55.2% respectively. In general, POC accounted for 24.5, 27.1 and 29.5% of the total SOC in OGR, CGE and GDE respectively.

**Table 3:** Distribution of particulate organic carbon with depth in three grazing systems in Chepareria, Kenya

Depth (cm)	Particulate organic carbon (g kg^−1^)			Particulate organic nitrogen (g kg^−1^)		
	OGR	CGE	GDE	OGR	CGE	GDE
0–10	1.40 ± 0.21Ca	2.01 ± 0.26Ba	2.28 ± 0.34Aa	0.19 ± 0.12Aa	0.16 ± 0.04Aa	0.16 ± 0.03Ab
10–20	1.20 ± 0.24Cb	1.52 ± 0.26Bb	1.80 ± 0.25Ab	0.17 ± 0.07Aa	0.18 ± 0.02Aa	0.18 ± 0.04Aab
20–40	0.88 ± 0.15Bc	1.31 ± 0.16Ac	1.32 ± 0.19Ac	0.18 ± 0.04Aa	0.18 ± 0.05Aa	0.20 ± 0.03Aa
Pooled mean	1.16 ± 0.30C	1.61 ± 0.37B	1.80 ± 0.50A	0.18 ± 0.07A	0.17 ± 0.05A	0.18 ± 0.04A

Values represent mean ± SD (n = 9). Values with different uppercase letters across the rows (grazing systems) and the lowercase letters within columns (soil depths) are significantly different at *P* < 0.05

*GDE* grazing dominated enclosure, *CGE* contractual grazing enclosure, *OGR* open grazing range

### Microbial biomass carbon and nitrogen

Enclosures significantly increased MBC and MBN, with higher concentrations observed in the 0–10 cm depth in all the grazing systems (*P* < 0.001, Table 4). Compared to the mean MBC recorded in OGR, the MBC contents in CGE and GDE significantly increased by 13.9% and 30.5% (*P* < 0.001). Within the enclosures, significantly higher concentration of MBC was observed in GDE relative to CGE (*P* = 0.01). However, MBC and MBN concentrations were similar across the enclosure age classes (*P *= 0.63 and 0.97 for MBC and MBN respectively).

**Table 4:** Distribution of microbial biomass carbon and nitrogen with depth in three grazing systems in Chepareria, Kenya

Depth (cm)	Microbial biomass carbon (µg g^−1^)			Microbial biomass nitrogen (µg g^−1^)		
	OGR	CGE	GDE	OGR	CGE	GDE
0–10	77.08 ± 5.25Ca	88.22 ± 6.16Ba	96.63 ± 5.31Aa	37.57 ± 2.01Ba	38.44 ± 2.26Ba	40.9 ± 5.68Aa
10–20	73.67 ± 4.27Cb	81.05 ± 3.74Bb	94.10 ± 5.55Aa	36.24 ± 2.50Aa	37.57 ± 3.45Ab	37.89 ± 3.30Ab
20–40	32.05 ± 7.25Cc	38.94 ± 10.42Bc	47.77 ± 6.04Ab	18.01 ± 3.71Cb	22.09 ± 3.04Ac	21.64 ± 3.34Ac
Pooled mean	60.93 ± 21.36C	69.40 ± 23.04B	79.50 ± 23.28A	31.97 ± 7.49B	31.34 ± 10.00B	33.48 ± 9.49A

Values represent mean ± SD (n = 9). Values with different uppercase letters across the rows (grazing systems) and the lowercase letters within columns (soil depths) are significantly different at *P* < 0.05

*GDE* grazing dominated enclosure, *CGE* contractual grazing enclosure, *OGR* open grazing rangeland

### Relationship between SOC, TN, POC and Microbial biomass

Total SOC exhibited significant (*P* < 0.001) positive correlation with TN, POC and MBC at all soil depths, but was only significant with PN at 10–20 cm depth (Table 5). Total nitrogen showed significant relationship with POC at all soil depths and with MBC at the surface 0–10 cm only. The POC positively associated with MBC at all soil depths with the relationship being stronger at the surface 0–10 cm (*r *= 0.63) compared to 10–20 and 20–40 cm depths (*r* = 0.57 and 0.41 respectively) (Table 5).

**Table 5:** Linear correlation analysis of SOC, TN, POC, PON, MBC and MBN in the three soil depths (n = 81)

Depth (cm)	SOC	TN	POC	PN	MBC	MBN
0–10						
SOC	–					
TN	0.71**	–				
POC	0.86**	0.70**	–			
PN	0.06	0.10	0.04	–		
MBC	0.57**	0.46**	0.63**	0.18	–	
MBN	0.32**	0.10	0.38**	0.01	0.21*	–
10–20						
SOC	–					
TN	0.54**	–				
POC	0.81**	0.42**	–			
PN	0.29**	0.14	0.25*	–		
MBC	0.40**	0.06	0.57**	0.15	–	
MBN	0.04	0.10	0.03	0.16	0.18	–
20–40						
SOC	–					
TN	0.66**	–				
POC	0.91**	0.65**	–			
PN	0.16	0.19	0.14	–		
MBC	0.30**	0.09	0.41**	0.10	–	
MBN	0.17	0.14	0.17	0.00	0.05	–

Values are correlation coefficient, *r*

*SOC* total soil organic carbon, *TN* total nitrogen, *POC* particulate organic carbon, *PON* particulate organic nitrogen, *MBC* microbial biomass carbon, *MBN* microbial biomass nitrogen

* Denotes significant correlation at the 0.05 level

** Denotes significant correlation at the 0.01 level: others are not significant

## Discussion

Similarities in soil pH, texture and CEC indicated that areas inside enclosures were comparable to the communal grazing lands prior to the establishment of enclosures and that differences in the measured variables among the studied sites were caused by land use change and not by inherent site variability. Low CEC indicated the deficiency of significant amounts of exchangeable cations such as Ca^2+^, Mg^2+^, and K^+^ [50]. Despite the fact that the top-soil bulk density in all the grazing systems were generally below the root-restricting value of 1.80 g cm^−3^ for loamy sand soils [51], the lower bulk density under GDE and CGE indicated the potential of enclosures to improve soil physical properties such as compaction that hamper critical soil functions, like the capture, storage and supply of water for plants [52]. This result agreed with [53] who showed that grazing exclusion sites reduced soil bulk density compared to the adjacent continuous grazing sites in the sandy grassland of Inner Mongolia, China. Higher soil moisture content in CGE and GDE could perhaps be as a result of the improved soil physicochemical properties. The reduced soil bulk density in the enclosed systems may have increased the rate of water infiltration in the soil due to high pore space. As indicated by [54], low water infiltration rates in degraded grasslands relative to enclosed sites were due to the high soil compaction induced by the grazing livestock. On the other hand, higher SOC in the enclosures increased the capacity of the soil to retain moisture [55]. Increase in moisture with depth may be due to high evaporative loss at the soil surface than in the deep soil horizons.

Irrespective of land use, the amounts of SOC and TN in soil are determined by the balance between organic matter inputs and losses [56]. The significantly higher level of SOC and TN in the enclosures compared to the open grazing land was probably because of the reduced soil disturbance by grazing animals. This prompted the production of aboveground biomass [36], thereby facilitating the accumulation and storage of C into the soil and its mineralization releasing nitrogen. According to [36, 53, 57], high removal of forage by the grazing animals in open grazing lands reduces herbaceous vegetation cover and accumulation of aboveground biomass. Consequently, this reduced the amount of C incorporated into the soil in open grazing lands. In addition, the high bulk density in the surface 0–10 cm and low soil moisture content in OGR could have reduced the input of soil organic matter by hampering storage and supply of water for plant growth [52, 54]. The reduction in SOC with increasing soil depth in all grazing systems suggests that organic matter accumulation in the surface 0–10 cm was higher than in the 10–20 cm and 20–40 cm depths. Higher SOC in the 10–20 cm and 20–40 cm in CGE and GDE relative to OGR could be as a result of the reduced grazing activities, which promoted root growth and accumulation of root biomass [33]. This facilitated the incorporation organic C in the subsoil. The reduction in SOC content with increasing soil depth is consistent with previous research in semi-arid rangelands in Tigray, Ethiopia and Inner Mongolia in China [7, 58]. These results corroborate with studies conducted in semiarid grasslands in Northern and Eastern Ethiopia and in Northwestern Kenya where higher soil organic C in enclosures was attributed to increased biomass production and reduced trampling by the grazing livestock [7, 8, 19]. Age of enclosure did not influence SOC levels because enclosures are continuously used for periodic grazing. This agrees with other studies in the area [59, 60]. Furthermore, the ~ 30 years of existence of enclosures in the area could be a short time to detect the changes in total organic carbon [61].

The higher concentration of POC in the enclosures suggested that the accumulation of organic matter was higher in the fenced areas than in the open grazing areas. Compared to total SOC, the considerably higher POC content in GDE than in CGE implied that POC is more sensitive to changes in grazing management. The results were consistent with [62, 63] who reported that particulate organic carbon responds to changes in grazing management compared to total SOC. Higher concentration of POC in GDE relative to CGE and OGR may be due to the lower grazing pressure which reduced soil disturbance. The reduced soil disturbance permitted the protection of soil organic matter from decomposition. According to [64, 65], trampling by livestock disintegrates soil macro-aggregates thus exposing soil organic matter to decomposition. The incorporation and stabilization of particulate organic matter into soil aggregates is a dominant factor for protecting organic carbon in grazing lands [66–68]. In addition, the higher herbaceous vegetation cover observed in CGE and GDE compared to OGR [36], greatly contributed to the conservation of POC in the enclosures by reducing erosion. Higher levels of POC in the surface 0–10 cm soil compared to 10–20 and 20–40 depths suggest that plant roots supplied more organic matter in the surface soil compared to the subsoil. The sandy nature of soils in this study (Table 1) implies that the POC have low colloidal protection, and consists mainly of partially humified plant residues. The proportion of POC of the total SOC in this study (24.5–29.5%) was within the reported ranges of between 2 and > 50% in semiarid grasslands [69–71].

Similar to the trends observed with POC, the significantly higher contents of MBC and MBN in the GDE and CGE compared to OGR was attributed to the increased concentration of POC in the enclosures which acted as a source of energy for soil microbiota. This was supported by the significant positive correlation exhibited between MBC and POC in all soil depths. Moreover, the significant decrease in MBC and MBN content with depth in all the grazing systems indicated a higher potential for organic matter inputs from root exudates and plant litter in the surface soil relative to the deeper soils [72]. These results were consistent with studies by Wu [6, 19] in a semi-arid rangeland in North-Western Kenya and Hulunbuir grassland of Inner Mongolia where higher microbial biomass C and N contents were recorded in enclosed areas than in the open grazing lands. The range of microbial biomasses C recorded in this study (32.1–96.6 μg g^−1^ soil) was relatively low compared to those recorded in Baringo County in Kenya (73–156 μg g^−1^ soil) [19]. This could be attributed to the differences in soil type and management strategies in the two areas. However, it has been recognized that microbial biomass recovers slowly in sandy soils in semiarid climates [64, 73]. Nonsignificant variations in POC and microbial biomass levels among the enclosure age classes could be the short residence time soluble fractions of organic C [74, 75].

## Conclusions

This study showed that the soils in the semi-arid rangelands of West Pokot County are very fragile. Relative to the enclosure systems, continuous grazing in the open grazing land caused a considerable increase soil bulk density and additional loss of total SOC, total N, POC, and microbial biomass contents. The observed variations in all these parameters indicated that the communal grazing lands were in a degraded state. This may portray serious consequence for soil quality, plant growth and loss of livelihood in tropical rangelands where grazing is the major land-use. Restoration of the degraded grazing land via the establishment of pasture enclosures increased the contents total SOC and total N and reduced soil bulk density. The concentrations of POC, MBC and MBN were considerably higher in GDE than in CGE. The results supported the hypothesis that GDE enclosures are more effective to restore degraded soils than CGE enclosures. This indicates that the degraded soils in the open grazing land can indeed recover following the establishment of enclosure. The POC and MBC were more sensitive to grazing management than total SOC and can be used as indicators of the soil C dynamics in semi-arid rangelands. Therefore, this study demonstrated that controlling livestock grazing through the establishment of enclosures is integral to increase SOC stocks or reduce its losses; a precondition for improving soil quality and climate change mitigation. Future research should focus on enclosures carrying capacity and seasonal ecosystem dynamics of carbon and nitrogen to better understand the ecology of this fragile ecosystem.

## Abbreviations

ANOVA: analysis of variance
BD: bulk density
CEC: cation exchange capacity
CGE: contractual grazing enclosure
DM: dry matter
EC: electrical conductivity
GDE: grazing dominated enclosure
LSD: least significance difference
MBC: microbial biomass carbon
MBN: microbial biomass nitrogen
OGR: open grazing rangeland
POC: particulate organic carbon
SOC: soil organic carbon
TN: total nitrogen
